# Biological Involvement of MicroRNAs in Proliferative Vitreoretinopathy

**DOI:** 10.1167/tvst.6.4.5

**Published:** 2017-07-10

**Authors:** Hiroki Kaneko, Hiroko Terasaki

**Affiliations:** Department of Ophthalmology, Nagoya University Graduate School of Medicine, Nagoya Japan

**Keywords:** microRNA, epithelial-mesenchymal transition, retinal pigment epithelium, retinal detachment, proliferative vitreoretinopathy

## Abstract

Even with a high surgical success rate for retinal detachment and proliferative vitreoretinopathy (PVR) supported by the robust improvement in vitrectomy surgery and its related devices, certain questions still remain for the pathogenesis and treatment of PVR. One of the important biological events in PVR is epithelial–mesenchymal transition (EMT) of the retinal pigment epithelial (RPE) cells. MicroRNAs are noncoding, small, single-strand RNAs that posttranscriptionally regulate gene expression and have essential roles in homeostasis and pathogenesis in many diseases. Recently, microRNAs also had a critical role in EMT in many tissues and cells. One main purpose of this brief review is to describe the knowledge obtained from microRNA research, especially concerning vitreoretinal diseases. In addition, the potential role of microRNAs in prevention of PVR by regulating EMT in RPE cells is described. Understanding microRNA involvement in PVR could be helpful for developing new biological markers or therapeutic targets and reducing the rate of visual disability due to PVR.

## Introduction

Vitrectomy surgery and related devices have improved greatly over the last few decades, and they enable us to reach higher successful rates after the surgeries for retinal detachment (RD). However, failure to reattach the retina sometimes causes the pathogenesis of proliferative vitreoretinopathy (PVR).^1–3^ Biologically, among many types of cells, for example, glial cells, fibroblasts, macrophages, and lymphocytes, involved in the pathogenesis of PVR,^4–6^ an important concept in PVR is epithelial–mesenchymal transition (EMT) of the retinal pigment epithelial (RPE) cells. In an eye with RD, RPE cells float into the vitreous cavity through the retinal breaks, adhere to the surface of the sensory retina, and undergo transformation from epithelial to fibrotic cells. Many types of growth factors and cytokines, such as platelet-derived growth factor, fibroblast growth factor (FGF), and epidermal growth factor, have been reported to be upregulated in the eyes with PVR; transforming growth factor β (TGFβ), a strong inducer of EMT, also is upregulated in the eyes with PVR and has a pivotal role in PVR.^4,7,8^ Glucocorticoid is a potent inhibitor of TGFβ signal; therefore, steroid treatment theoretically is potent in preventing PVR.^9,10^ However, previous studies showed insufficiency of the corticosteroid treatment; thus, an additional approach is required urgently.^11–13^ On the other hand, the explosion of biological knowledge about noncoding RNA has shed light on the importance of microRNAs in many diseases. Over the last several years, the involvement of microRNAs in the pathogenesis of PVR has been elucidated. In this brief review, we discussed previous studies that have shown the biological importance of microRNAs in the pathogenesis of PVR, especially focusing on EMT of RPE cells. Lastly, we shared new data based on our recent findings on the biological relationships between microRNA and inflammatory cytokines and caveolin-1.

### RD and PVR

**Figure 1:**
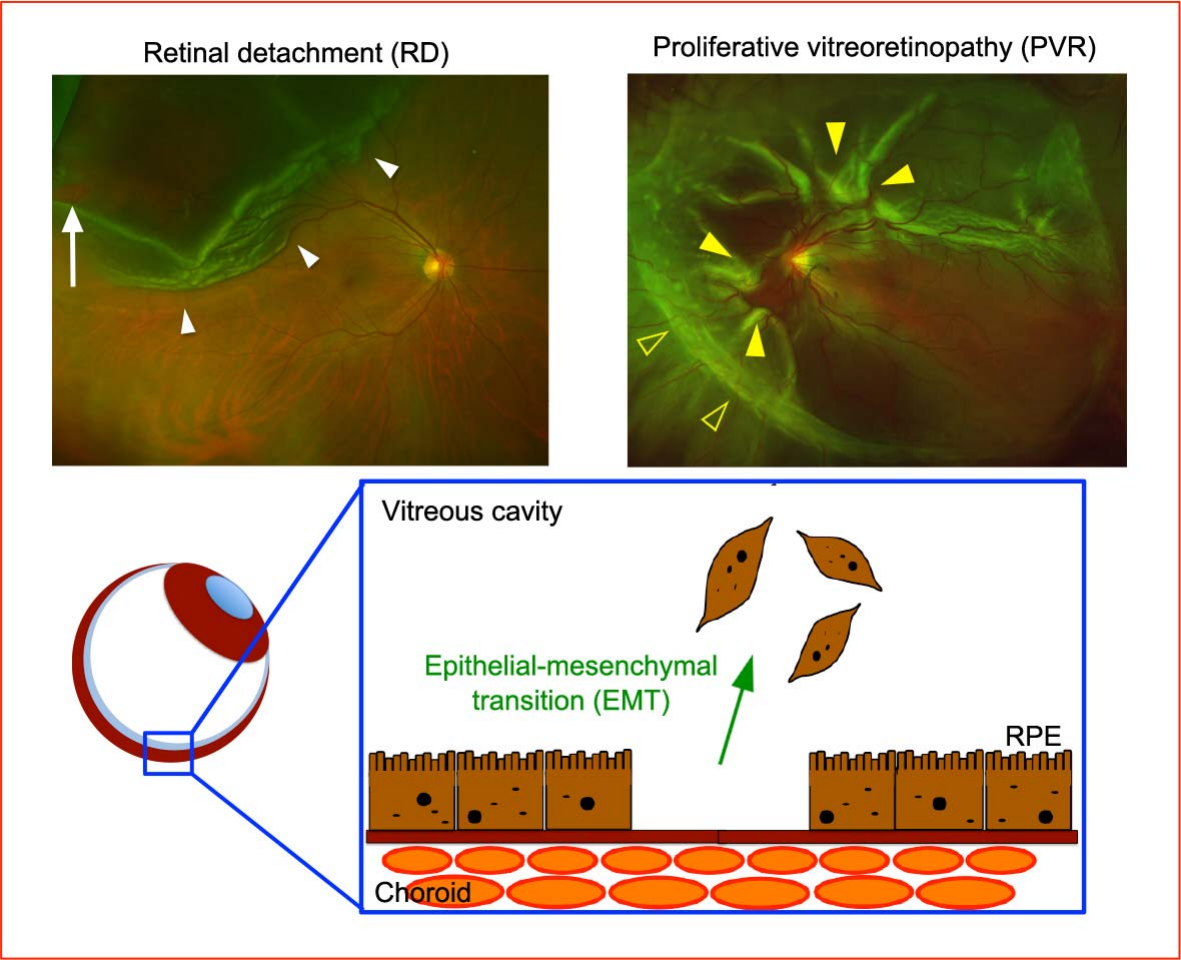
Illustration of pathogenesis of PVR. In eyes with RD (*left*), floating RPE cells receive certain biological signals, induce epithelial–mesenchymal transition, and RPE-derived fibrotic cells migrate on the surface of the retina. Color fundus image of PVR (*right*) showing wrinkling of the retinal surface, retinal stiffness, vessel tortuosity, and subretinal strands. The *white arrow* indicates a retinal break, and the *white arrowheads* indicate detached retina. The *yellow filled arrowheads* indicate wrinkling of the retinal surface, and *yellow open arrowheads* indicate epiretinal fibrotic membranes.

The incidence of RD has been reported to be as low as 0.6 to 1.8 per 10000 people.^14^ The most common type of RD is rhegmatogenous RD, which is caused mainly by retinal breaks owing to vitreous traction.^15^ Although vitrectomy surgery has contributed greatly to the successful treatment of severe retinal diseases, including RD, it is difficult to recover all cases of RD completely. Patients with severe and/or long-standing RD or those with unsuccessful surgical treatment tend to suffer PVR.^16,17^ PVR is diagnosed clinically by the following clinical observations:^18^ existence of vitreous haze, vitreous pigment clumps, pigment wrinkling of the retinal surface, retinal stiffness, vessel tortuosity, rolled and irregular edge of retinal breaks, and subretinal strands. In an eye with RD, RPE cells float into the vitreous cavity through retinal breaks, adhere to the surface of the sensory retina, and undergo transformation from epithelial to fibrotic cells. This biological phenomenon is referred to as EMT, in which differentiated RPE-derived cell help induces PVR (Fig. 1).^19–22^

### MicroRNA

Even after robust breakthroughs enabled by the whole human genome project, dozens of diseases remain of which the pathogeneses have not been elucidated perfectly. Unexpectedly, only 2% of the human genome is responsible for coding proteins.^23^ Scientific approaches increasingly have begun to use transcriptome analysis. Scientists have recognized that thousands of noncoding RNAs are transcribed in the human body. One of the important but underestimated noncoding RNAs is microRNA. MicroRNA is an extensive class of endogenous, noncoding, single-strand RNAs with 18 to 24 nucleotides that negatively regulate gene expression by interacting with the 3′-untranslated regions (3′UTR) of their target mRNAs.^24^ By modulating the expression of their target genes, microRNAs have essential roles in homeostasis and pathogenesis.^25–27^ In the human body, more than 2000 microRNAs reportedly are involved in cell proliferation, differentiation, and signaling. These microRNAs regulate cellular processes, including tumor formation, and have been linked to a number of human diseases; thus, the role of microRNA as a therapeutic target or a disease marker has been an active area of research.^28–31^ In the eye, various microRNAs are thought to act on the retina or on RPE cells and to have important roles in neuroprotection and angiogenesis.^20,32–35^

### Publications in MicroRNA and Vitreoretinal Diseases

**Figure 2:**
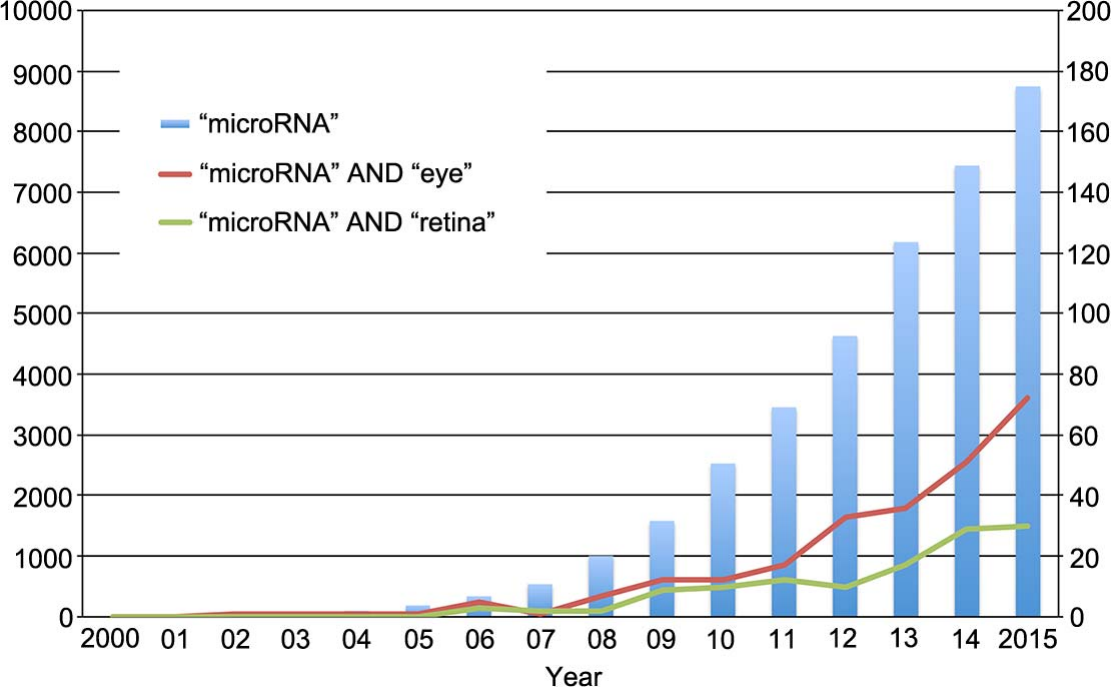
Growth of the number of microRNA-related publications. PubMed entries that reference the term “microRNA” are represented by the *blue bars*, those that reference “microRNA” AND “eye” are represented by the *red line*, and those that reference “microRNA” AND “retina” are represented by the *green line*.

The number of publications that have described microRNA experiments and ocular diseases has been increasing (Fig. 2). Soon after the first use of the term “microRNA” in the literature in 2001,^36–38^ articles in PubMed referencing the keywords “microRNA” AND “eye” appeared in 2001. In 2006, articles in PubMed referencing the keywords “microRNA” AND “retina” appeared (Fig. 2). As scientific technology and knowledge of microRNA has increased, the number of microRNA-related publications in ophthalmic research also has increased each year. Table 1 lists the published studies on microRNAs in retinal diseases, including the targeted microRNAs and the cells/animals.

**Table 1:**
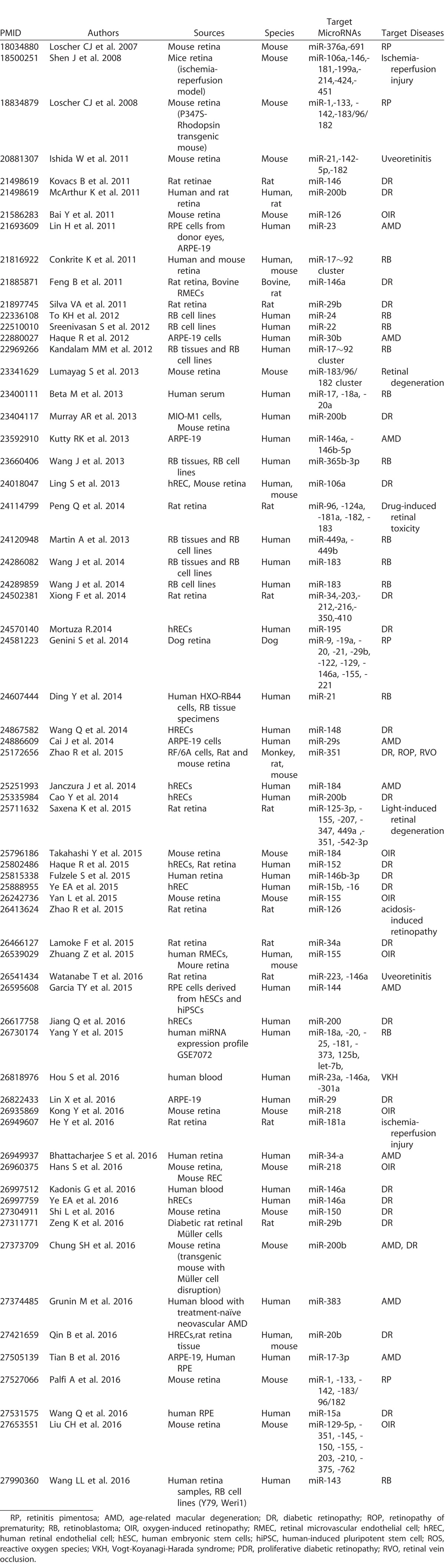
List of Published Studies on MicroRNA in Retinal Diseases

### Detecting MicroRNAs in the Vitreous from Human Eyes

In eyes with RD or PVR, higher expressions of inflammatory cytokines have been detected in the vitreous,^39–41^ presumably because a variety of factors are eluted from retinal cells, RPE cells, and vitreous cells into the vitreous. In addition, RD and PVR implicate the breakdown of blood–retinal barriers; therefore, allowing the passage of cells into the retina and vitreous cavity.^42,43^ These factors have the potential to have biological effects on many intraocular cells. The vitreous occupies the cavity of the posterior segment of the eye and is in direct contact with the retina. In addition, the vitreous also is in direct contact with the RPE through the retinal breaks. Therefore, in eyes with RD and PVR, ectopic RPE cells float in the vitreous. Examining microRNA expression in the vitreous in patients with a targeted vitreoretinal disease is a great tool for understanding the relationship between microRNAs and vitreoretinal disease. Table 2 lists the previous publications of studies that have analyzed microRNAs from surgically collected human vitreous.^44–49^ In most studies, microRNAs were detected by microRNA arrays, followed by reconfirmation by quantitative real-time polymerase chain reaction assay. Almost all of the current surgical instruments used in vitrectomy surgery are disposable and RNase-free, and collecting the vitreous from patients without any additional risks is possible during the regular surgical procedure. It is presumed that the number of studies involving collecting and analyzing microRNA in the vitreous will increase.

**Table 2:**
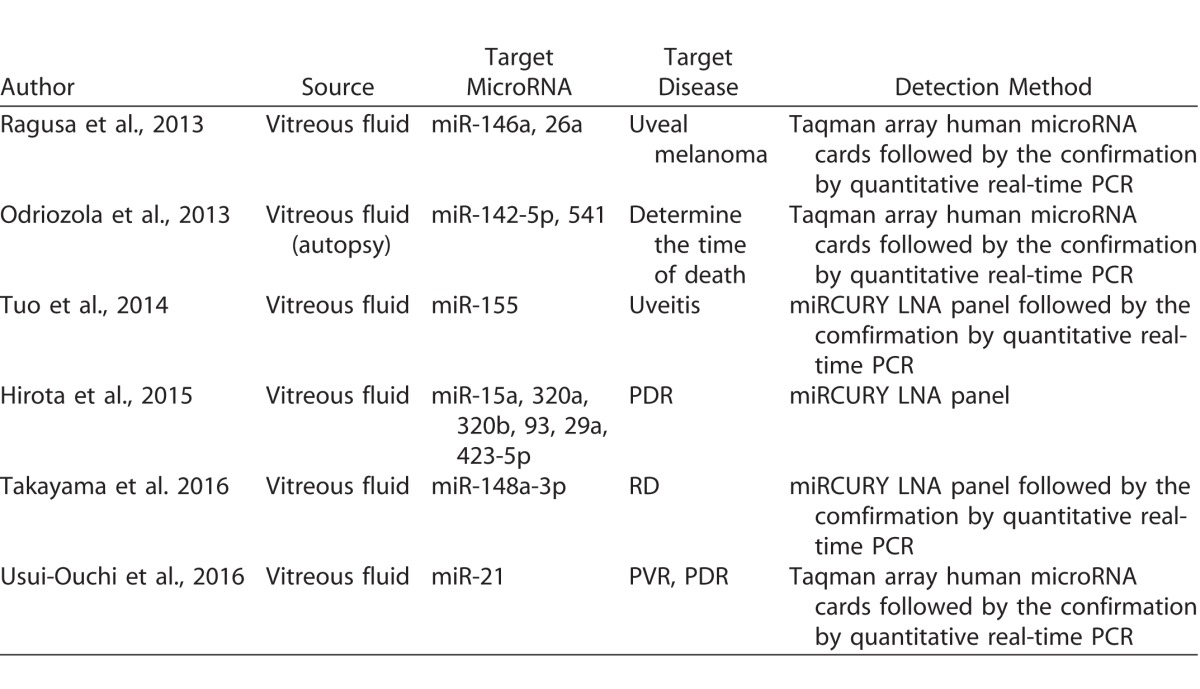
List of Published Studies on MicroRNA in Human Vitreous Fluid

### MicroRNA and EMT in RPE Cells

Biological evidence has been established showing that specific microRNAs induce/inhibit EMT in other cells.^50–52^ Recently, aberrant expression of microRNAs in RPE cells undergoing EMT was reported.^47,49,53–59^ RPE cells undergo EMT and contribute to the pathogenesis of PVR. We focused on previous studies that have examined the effect of microRNAs on human RPE cells via EMT. Table 3 shows the list of publications that have examined the biological interactions between microRNA and EMT-related factors; for example, TGFβ. Chen et al.^55^ reported that 185 microRNAs were downregulated and 119 microRNAs were upregulated in TGFβ2-treated ARPE-19 cells, which suggested that microRNAs have important roles in EMT of RPE cells. Li et al.^57^ revealed that miR-29b is downregulated and has a significant role in TGFβ1-induced EMT of ARPE-19 cells. Overexpression of miR-29b prevents progression of EMT by targeting AKT2, a noncanonical signaling pathway in TGFβ-induced EMT. Wang et al.^58^ showed that miR-204/211 preserves the epithelial phenotype in primary human fetal RPE (hfRPE) cells, indicating a pivotal role of miR-204/211 in EMT of hfRPE cells. A large number of genes and proteins regulate EMT in RPE. Most microRNAs regulate the transcription of these EMT-related factors and indirectly regulate EMT in RPE cells.

**Table 3:**
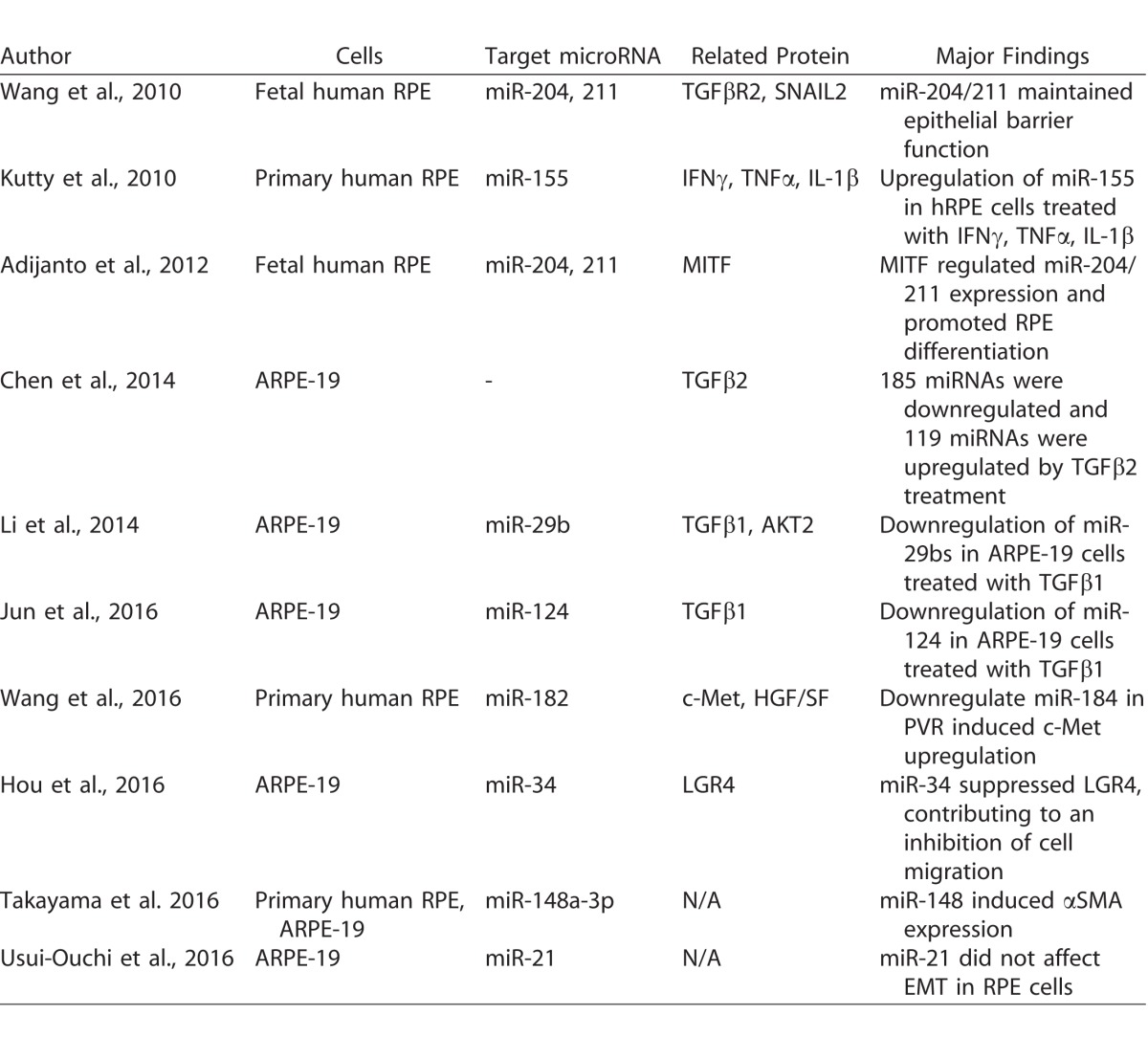
List of Published Studies on MicroRNA and Cultured RPE Cells

### MicroRNA and Other Factors in PVR

**Figure 3:**
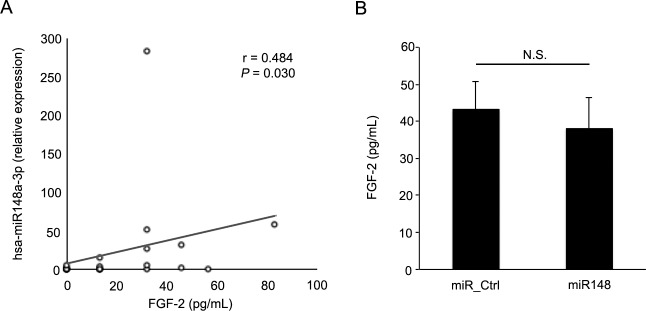
Correlation between microRNA expression and inflammatory cytokines. (A) miR-148a expression in eyes with RD was significantly correlated with FGF-2, but not with IFN-γ, IL-10, IL-12p40, IL-1β, IL-6, IL-8, MCP-1, TNFα, and VEGF. (B) FGF-2 levels that was secreted in the medium from hRPE cells transfected with miR148_mimic did not show significant difference from those with control microRNA (*n* = 12, *P* = 0.54).

**Figure 4:**
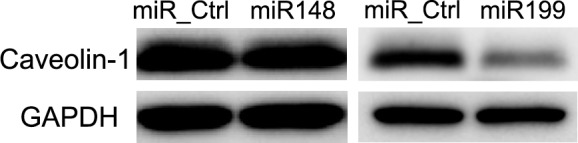
Caveolin-1 expression is altered by miR-199, but not by miR-148a. Shown is a comparison of caveolin-1 expressions in primary human RPE cells after being transfected with miR148-mimic versus control microRNA (miR_Ctrl) and with miR199-mimic versus control microRNA (miR_Ctrl). Caveolin-1 is suppressed by miR-199, but not by miR-148a.

A large number of cytokines, such as EGF and TGF-β, induce EMT. In addition, TGF-β cooperates with other cytokines, such as FGF, and enhances EMT effectively.^60–62^ For instance, TGF-β has been shown to induce isoform switching of FGF receptors, which sensitizes the cells to FGF-2 and suggests that TGF-β and FGF-2 cooperate with each other and regulate EMT.^61^ Recently, we found that miR-148a was expressed specifically in the vitreous from eyes with RD and that miR-148a expression correlated with the size of the retinal breaks and duration of RD, which has been reported to be the possible cause of PVR.^47,63^ Based on the aforementioned reports showing that the vitreous has multiple upregulated inflammatory cytokines, we examined correlations between miR-148 expression and inflammatory cytokines from eyes with RD. Demographic and clinical characteristics, and the results of inflammatory cytokine levels of the 20 patients with RD are listed in Table 4. miR-148a expression in the vitreous was correlated significantly with FGF-2 expression levels in the vitreous of eyes with RD; however, FGF-2 expression secreted from hRPE cells transfected with miR-148a mimic did not significantly differ from that of cells transfected with control microRNA in vitro (Fig. 3). In the meantime, our recent study found that caveolin-1, an integral membrane protein, was upregulated in proliferative membranes from eyes with PVR and had a pivotal role in suppression of EMT in RPE cells.^64^ Therefore, we investigated whether specific microRNAs have biological relationships with the expression of caveolin-1 in RPE cells. The microRNAs that were detected specifically in the vitreous and subretinal fluid from the eyes with RD were listed in our previous study.^47^ In addition, Table 5 lists the microRNAs that were detected specifically in the subretinal fluid. Using miRBase and microRNA.org databases,^65,66^ we hypothesized that miR-199a-5p is a possible regulator of caveolin-1. We found that caveolin-1 was suppressed by miR-199a-5p but not by miR-148a (Fig. 4). These results, which corroborated those obtained from our previous study, indicated that higher expression of miR-148a in the vitreous may contribute to the pathogenesis of PVR by an unknown mechanism and miR-199a-5p in the subretinal fluid may have a role in the pathogenesis of PVR by regulating caveolin-1 expression in RPE cells.

**Table 4:**
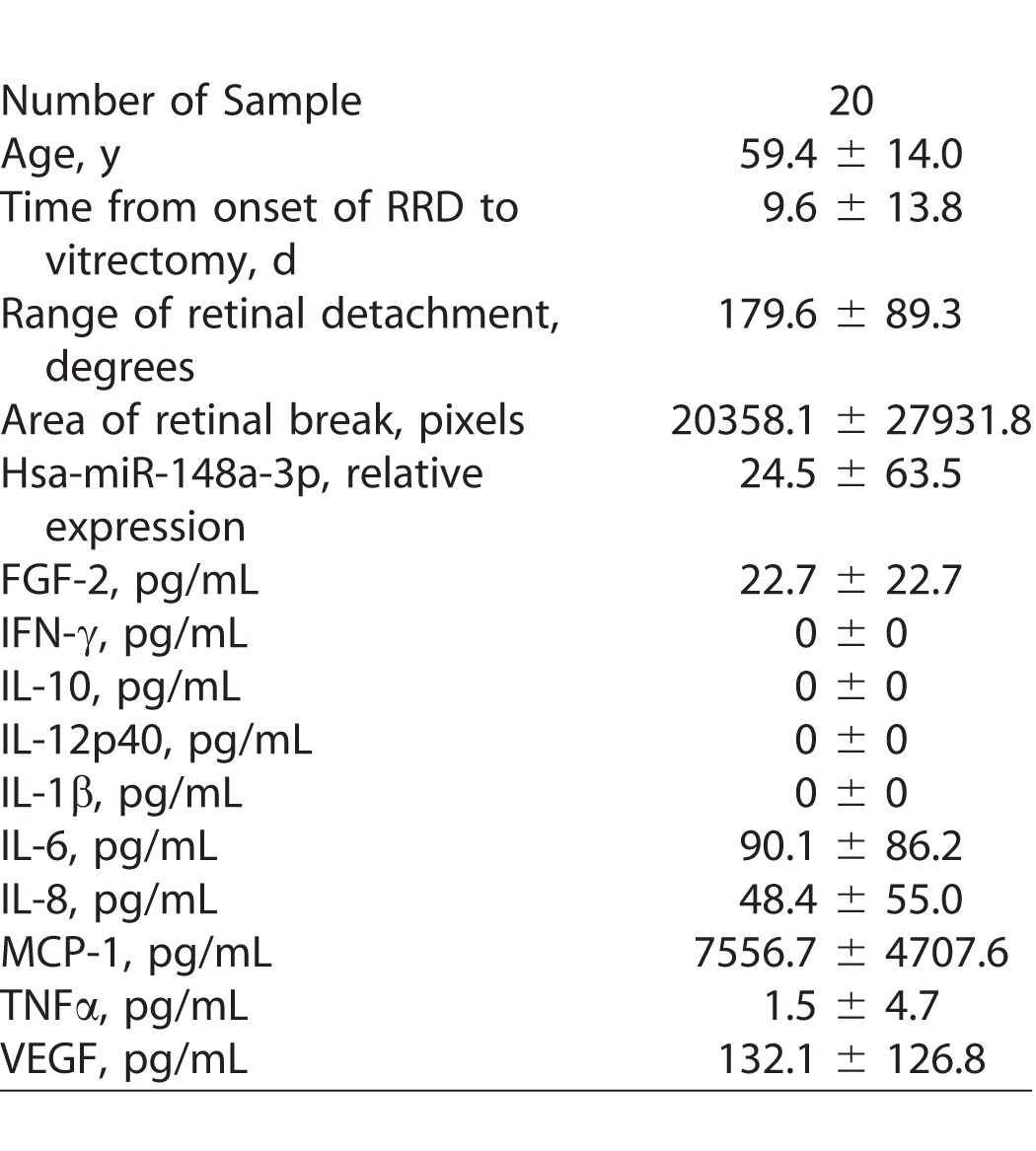
Demographic and Clinical Characteristics and the Biological Parameters of the Patients with RD

**Table 5:**
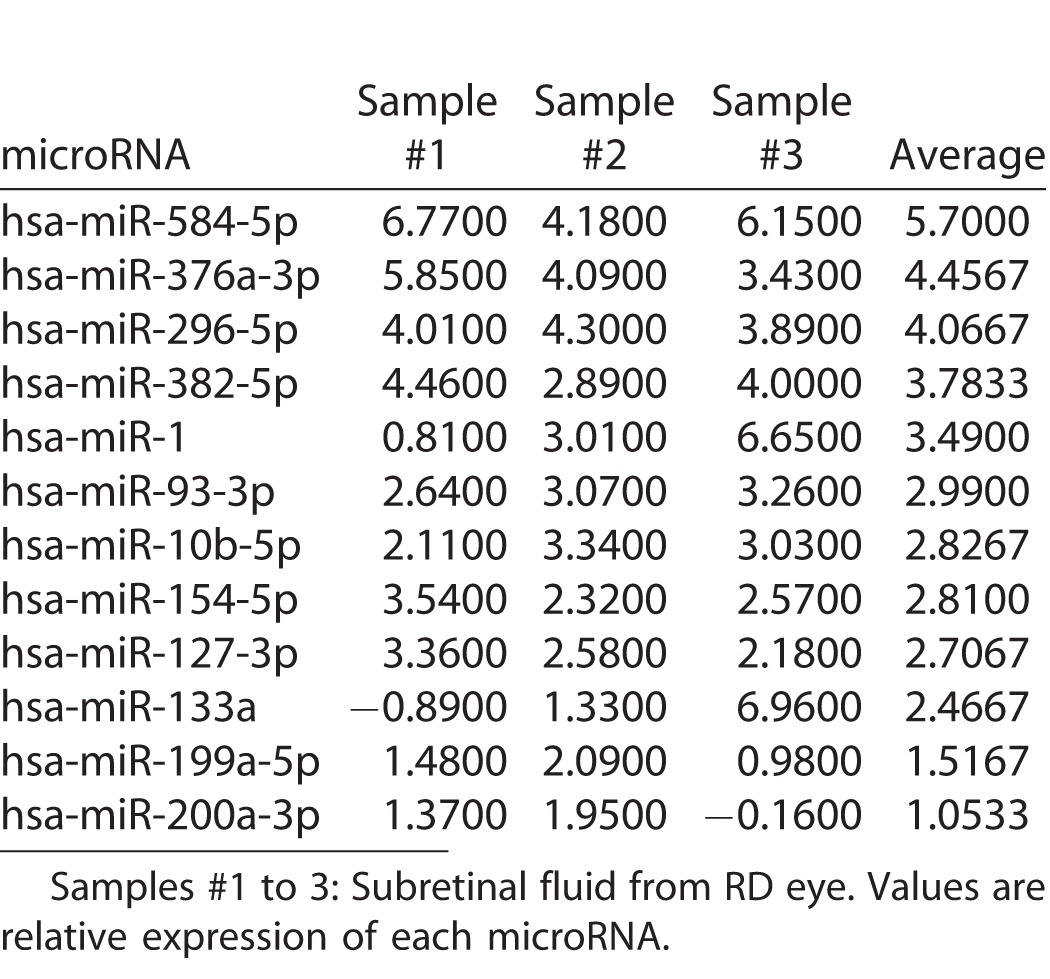
List of MicroRNAs Detected Specifically in the Subretinal Fluid

## Discussion

Due to the developments of surgical devices and biological understanding, the success rate for structural recovery of PVR has been improved. Nevertheless, a certain number of patients with PVRs become blind even after thorough surgical and medical intervention. Achieving understanding of the pathologic mechanisms underlying the development of fibrotic membrane spreading on the surface and beneath the sensory retina is critical to completely overcome PVR. MicroRNA is a novel and powerful class of modulators that regulate gene expression, and its involvement in the pathogenesis of PVR now is becoming clear. Over the last few decades, biological tools to examine inflammatory cytokines have revolutionized research capabilities. Consequently, the involvement of inflammatory cytokines in many retinal diseases also has been elucidated. In addition to further development of scientific tools for microRNA detection, measurement, and functional analysis, the close relationship of microRNA and cytokines must be elucidated. Identifying the role of other microRNAs in EMT in RPE cells in vitro and in PVR in vivo could be helpful for understanding more precisely the involvement of EMT-related gene expression. In this brief review, we showed the lists of microRNAs that reportedly were relevant to retinal diseases, including PVR. Subsequently, based on our previous studies, we proposed two microRNAs that possibly have important roles in PVR. There might be a difficulty in constructing efficient experimental design for microRNA research: in our case, we first targeted miR-148 because it was expressed most abundantly in the vitreous of RD.^47^ We found miR-148 induced EMT in RPE cells. However, we could not find a specific gene that affects EMT of RPE cells under miR148 regulation. In the meantime, we found the role of caveolin-1 in EMT, and we also found miR199 is a possible regulator of caveolin-1. Finding a set of microRNA and its targeted gene in the same specific disease is the key to exploring microRNA-based therapeutic possibility. Further improvement of the microRNA research will accelerate the development of microRNA agonists/antagonists that can be used as a new class of drugs to regulate the progression of PVR.

## Materials and Methods

### Patients with Retinal Detachment and Sample Collection and Analysis

The methods for sample collection have been described in our previous studies.^47,63^ Briefly, all vitreous samples were collected by dry vitrectomy and subretinal fluid was collected during scleral buckling surgery and immediately stored at −80°C. The vitreous was thawed and centrifuged for 5 minutes at 2000*g*, 4°C to remove contaminating cells only once before performing the MILLIPLEX MAP Human Cytokine/Chemokine Panel (Merck Millipore, Billerica, MA), a bead-based multiplex immunoassay that allows simultaneous quantification of the following human cytokines: FGF-2, IFN-γ, IL-10, IL-12p40, IL-1β, IL-6, IL-8, MCP-1, TNFα, and VEGF. Values under the detection limits were defined as “0” in the statistical analyses. Our adhered to the guidelines of the Declaration of Helsinki; the study was approved by the Nagoya University Hospital Ethics Review Board. Written informed consent was obtained from all patients before their enrolment. Spearman's rank correlation coefficient was used to assess the association between expression levels of hsa-miR-148a-3p and cytokines in the vitreous. *P* < 0.05 were considered a statistically significant correlation.

### MicroRNA Mimic Transfection

The methods for the microRNA mimic transfection and sample collection have been described in our previous studies.^47,63^ Briefly, primary hRPE cells (Lonza, Walkersville, MD) were transfected with 60 pmol of miR-148a-3p mimic and miR-199 mimic (Invitrogen, Carlsbad, CA) before use in further in vitro experiments. hRPE cells were cultured in serum-free antibiotic-free Dulbecco's Modified Eagle's Medium premixed with Ham's F-12 (1:1 ratio; Sigma-Aldrich Corp., St. Louis, MO,) before incubation with Lipofectamine RNAiMAX Transfection Reagent and miR-148a-3p mimic for 48 hours. hRPE cells also were transfected with negative control mimic (miR_Ctrl) in the same manner and used as controls. Culture medium then was replaced with fresh medium containing 10% FBS and antibiotics and used in further experiments.

### Western Blotting and ELISA

Following hsa-miR-148a-3p mimic, hsa-miR-199 mimic, or miR_Ctrl transfection and TGF-β2 stimulation, hRPE cells were washed with PBS 3 times and then lysed in RIPA buffer (Sigma-Aldrich Corp.) containing a protease inhibitor cocktail (Roche Diagnostics, Ltd, Mannheim, Germany). The lysate was centrifuged at 15,000*g* for 15 minutes at 4°C, and the supernatant was collected. The protein concentrations were determined using a Bradford assay kit (Bio-Rad, Hercules, CA) with bovine serum albumin as the standard. Protein samples (20 μg) from human tissues or culture cells were run on 5% to 20% sodium dodecyl sulfate precast gels (Wako, Tokyo, Japan) and transferred to polyvinylidine fluoride (PVDF) membranes by using an iBlot blotting system (Invitrogen). Transferred membranes were washed in 0.05 M Tris, 0.138 M NaCl, 0.0027 M KCl, pH 8.0, 0.05% Tween 20 (TBS-T; Sigma-Aldrich Corp.) and then blocked in 5% nonfat dry milk/TBS-T at room temperature (RT) for 2 hours. Membranes then were incubated with anti-caveolin-1 antibody (catalog no. 3238: 1:200; Cell Signaling Technology, Beverly, MA) at 4°C overnight. Protein loading was assessed by immunoblotting using an anti-GAPDH antibody (catalog no. 2118: 1:3000; Cell Signaling Technology). Membranes then were incubated with horseradish peroxidase-linked secondary antibody (catalog no. 7074: 1:3000; Cell Signaling Technology) for 1 hour at RT. Signals were visualized by enhanced chemiluminescence (ECL plus; GE Healthcare, Piscataway, NJ) and captured by using ImageQuant LAS-4000 (GE Healthcare). For measuring FGF-2 levels in the medium from hRPE cells, human FGF basic Quantikine ELISA Kit (catalog no. DFB-50; Minneapolis, MN) was used.
